# Combined effect of diabetes and frailty on mortality among Chinese older adults: A follow-up study

**DOI:** 10.3389/fendo.2022.1105957

**Published:** 2023-01-16

**Authors:** Jing Shi, Yongkang Tao, Lixiang Wang, Shuqiang Chen, Ziyi Zhou, Li Meng, Baiyu Zhou, Chunbo Duan, Huan Xi, Pulin Yu

**Affiliations:** ^1^ Beijing Institute of Geriatrics, Beijing Hospital, National Center of Gerontology, National Health Commission, Institute of Geriatric Medicine, Chinese Academy of Medical Sciences, Beijing, China; ^2^ Department of Gastroenterology, China-Japan Friendship Hospital, Beijing, China; ^3^ Department of Health Policy and Management, International University of Health and Welfare, Tokyo, Japan

**Keywords:** frailty, diabetes, mortality, elderly, follow-up study

## Abstract

**Background:**

Frailty and diabetes are two important health problems associated with aging in older individuals. This paper seeks to analyze the frailty in older adults suffering from diabetes and the combined effect of diabetes and frailty on mortality risk.

**Methods:**

The frailty index (FI) model was employed when evaluating frailty among the older adults based on the baseline data conducted in 2009; and death as outcome variables collected in 2020 were analyzed. The influence of diabetes on age-related changes in frailty in the older adults and resulting mortality rates was analyzed. Cox regression and Kaplan-Meier curves were applied to evaluate the influence on the risk of death and the 11-year survival of the older adults with varying diabetes and frailty statuses.

**Results:**

Ultimately, 1,213 older people aged between 60 and 101, with an average age of (74.79 ± 8.58) at baseline, were included in the analysis. By 2020, there had been 447 deaths with mortality at 36.9% (447/1,213); there were 271 cases of diabetes, with a prevalence of 22.3% (271/1,213). The mean FI value for older adults with diabetes was higher than that of those without regardless of age, and the average annual relative growth rate of the FI value for older adults with diabetes was higher than that of those without diabetes (*β =* 0.039 *vs. β =* 0.035*, t =* 8.367, *P <* 0.001). For all FI value levels, the mortality rate among older adults with diabetes was higher than that of those without. The Cox Regression analysis showed that, compared with those suffering from neither diabetes nor frailty, older adults with both had the higher mortality risk (*HR* = 1.760. P *<* 0.001), followed by older adults suffering from frailty alone (*HR* = 1.594, *P* = 0.006), and then by older adults suffering from only diabetes (*HR* = 1.475, *P* = 0.033). The survival analysis showed that the median survival of those suffering from diabetes and frailty to be the shortest at just 57.23 (95% *CI*: 54.05 to 60.41) months, lower than the 83.78 (95% *CI*: 79.33 to 88.23) months in those suffering from frailty alone, and 119.93 (95% *CI*: 113.84 to 126.02) months in those with only diabetes, and 124.39 (95% *CI*: 119.76 to 129.02) months in older adults with neither diabetes nor frailty (*P <* 0.001).

**Conclusion:**

Frailty is common among older adults suffering from diabetes, and there is an increased risk of poor health outcomes, such as death, among older adults suffering from diabetes and frailty. When diagnosing, treating, and dealing with older adults with diabetes, attention should be paid to screening and assessing frailty in hopes of identifying it early so that appropriate measures of intervention can be taken to avoid or delay the resulting adverse effects.

## Introduction

The ageing of the population has been accompanied by a dramatic increase in the prevalence of diabetes (1). Diabetes is associated with a variety of complications that include cardiovascular disease, retinopathy, renal failure, and peripheral vascular disease, all of which are capable of seriously affecting quality of life in older adults. China ranks first in diabetes, with more than 140 million patients suffering from the disease in 2021, and cases expected to rise to 174 million by 2045, of which 30% are older adults (2). Studies have shown diabetes to be accompanied by complications, disability, and something known as frailty syndrome (3). Frailty syndrome is characterized clinically by declined physiological reserve, multiple system disorders, increased susceptibility to internal stress, and decreased internal stability. Diabetes has been found to be a risk factor for frailty, and the two have been shown to interact with one another: on the one hand, long-term imbalance in blood glucose regulation increases protein and fat decomposition, and decreases muscle mass and strength resulting in frailty with such manifestations as fatigue and body mass reduction, while complications from diabetes also reduce immunity and mobility, ultimately leading to the development of frailty; on the other hand, frailty threatens to change glucose-insulin metabolism, while reduced energy intake and malnutrition aggravate the risk of hypoglycemia, to say nothing of the impact on the selection and treatment of blood glucose control drugs (4, 5). In comparison to the older adults not suffering from diabetes, older adults with diabetes experience an increased risk of frailty of approximately 60% (6); in addition, older adults suffering from diabetes and frailty tend to experience serious adverse health outcomes that include higher rates of mortality, disability, and readmission and a significant decrease in daily activities (3, 7). Consequently, the early identification of frailty and subsequent interventions in patients with diabetes are important for helping to avoid or delay adverse effects.

Studies have shown frailty to be an important factor in influencing blood glucose management in older adults suffering from diabetes (8). The International Position Statement on the Management of Frailty in Diabetes Mellitus emphasizes the importance of including the identification and assessment of frailty in the routine management of patients suffering from diabetes (9, 10). In developed countries, researchers attach importance to the monitoring, evaluation, and prevention of frailty in patients with diabetes and have carried out a large number of studies on how diabetes can be complicated by frailty. In China, older adults with diabetes can be found more and more often in the community, and so the management of their health has become a focus of many community health services; however, frailty has still yet to be included in routine screening for them (11). As of now, there continue to be few studies on the situation of older adults with diabetes and frailty in Chinese communities and on the influence of diabetes with frailty on long-term risks of mortality. This study chose to focus on the older adults in the urban communities of Beijing and to analyze the prevalence of diabetes and frailty in the older adults and its influence on the risk of mortality as a basis for the management of frailty among the older adults with diabetes and corresponding measures to reduce the resulting adverse health effects.

## Materials and methods

### Survey sites and subjects

This is a secondary analysis of the Health Status and Fall Status Follow-up Survey database, a representative cohort of urban community dwelling elder people aged 60 years and older in Beijing. In this study, the baseline survey population in 2009 was used as samples, and death events from this cohort collected in the follow-up survey in 2020 were used as the outcome variables. The baseline survey was conducted in 2009 in a community under the jurisdiction of a sub-district office in Dongcheng District, Beijing. In 2009, the proportion of elder people aged 60 years and older in this community was similar to that in the whole country at that time (13.9% vs. 12.5%), which could well represent the situation of the older adults in China (12). A total of 4 community neighborhood committees were randomly selected from the sub-district office, after which older subjects aged 60 years and older under the jurisdiction of the selected communities were selected on the basis of random cluster sampling for further analysis. Criteria for being included: resident individuals aged 60 years and older in the surveyed communities. Criteria for being excluded: individuals suffering from extreme frailty and unable or unwilling to complete the questionnaires. A total of 1,578 older adults met the survey requirements for 2009 as baseline samples. During the survey, 37 older adults refused to be interviewed, and 63 older adults could not be followed-up (could not be found in two visits during the survey), as a result of which 1,478 older adults were included in the end. By 2020, 232 older adults could not be followed-up, accounting for 15.7% (232/1,478), either as a result of having left or moved from the locale of the survey. Among the older adults who could not be followed-up, 108 (46.6%) were male and 124 (53.4%) were female, with an average age of (68.24 ± 3.58) years. In the end, follow-up data were available for 1,246 older adults, including 519 males (41.7%) and 727 females (58.3%), with an average age of (72.05 ± 4.52) years. Although the average age of those who could not be followed-up is lower than that of the older individuals included in the study (*t =* 12.148, *P <*0.001*)*, the gender difference between these two population is not statistically significant *(χ^2^ =* 1.921, *P* = 0.166). This study was approved by the ethics committee of Beijing Hospital (No.2020BJYYEC-134-02). All subjects signed the informed consent form.

### Survey content

This study used validated standard questionnaires selected following several rounds of expert discussion to investigate the target population, covering such content as demographic characteristics (age, gender, educational level, marital status), family support (whether they lived alone and whether they enjoyed positive family relations), social support [number of friends able to offer support (help), frequency of participation in group activities)], economic level, lifestyle (smoking, alcohol consumption, exercise), health and physical performance status (vision and hearing, walking balance), diseases (including diabetes) and medications, activities of daily living (ADL) and instrumental activities of daily living (IADL) (13), cognition and emotion [memory loss, emotional instability, Mini Mental State Examination (MMSE)] (13), depression [The Center for Epidemiological Studies Depression Scale (CES-D)] (13), and comprehensive geriatric assessment (falls, urinary incontinence, pain, constipation, weight loss, sleep disorder, usage of sleep aids), etc. Diseases needed to be diagnosed by a hospital at or above the county level, while those experience symptoms deemed to be subjective and lacking definite diagnosis were not included in the statistics.

### Assessment of frailty

The frailty index (FI) model developed by a team led by Professor Kenneth Rockwood, a Canadian geriatric expert, was used to quantitatively describe degree of frailty on the basis of the accumulation of health deficits (14). The FI calculation formula consists of the number of health deficits present in an individual/the total number of items considered health deficits. The FI value ranges between 0 and 1, with larger values indicating more serious degrees of frailty (15). Based on the content of the survey questionnaires, a total of 36 variables were selected as health deficit items according to the conditions for constructing the FI health deficit variables (15), including comprehensive geriatric assessment (7 items), vision and hearing (2 items), walking balance function (6 items), disease and medication (15 items), activities of daily living (2 items), cognition and emotion (3 items), and depression (1 item). Meanwhile, each variable was assigned a value according to its type. See Appendix 1 for each specific variable and its assignment. Using the grading method recommended by Searle et al. (15), a frail individual was defined as one having a FI of 0.2 or more.

### Definition of follow-up outcomes

Mortality among the follow-up survey subjects was used as outcome variable and included death (yes or no) and time to death. Information concerning death was collected or obtained by staff through the relatives of each subject, local neighborhood committees (for those without relatives), or local public security organs (for those without relatives and not belonging to any neighborhood committee). A precise method was used to calculate follow-up duration. If a subject died during the follow-up, the follow-up duration was calculated as (death date - baseline date)/12; if a subject was still alive, it was calculated as (last follow-up date - baseline date)/12.

### Statistical methods

SPSS 24.0 and Matlab 2020 software were used for data analysis and plotting, and any missing data values were imputed by Markov Chain Monte Carlo (MCMC) method, a multiple imputation method (16). Shapiro-Wilk test was used to check normal distribution for continuous variables. The normally distributed continuous variables were expressed as x ± s, an independent sample t*-test* was used for comparison between two groups, and analysis of variance (ANOVA) was used for comparison among multiple groups; non-normal variables were expressed as median and quartile *[M(Q1,Q3)]*, and Kruskal-Wallis *H* test was used for comparison among multiple groups; enumeration data were expressed in the number (or percentage) of cases, and *χ^2^* test was used for comparison between groups; nonlinear regression techniques were used to fit age-specific frailty index values as a function of age (an exponential function) and to fit the probability of death as a function of the frailty index (a logistic function) between older adults with and without diabetes*;* the Cox multivariate regression model was used to evaluate the hazard ratio (HR) of diabetes (yes or no) and frailty (yes or no) on the death of older adults, the Kaplan-Meier method was used to plot the survival curve to analyze the influence of different diabetes and frailty statuses on the survival time of older adults, and the log-rank method was used for testing. *P* < 0.05 was considered statistically significant.

## Results

### Comparison of baseline status of the older adults with or without diabetes and/or frailty

Of the 1,246 older individuals involved, 33 individuals suffering from subjective symptoms and lacking a definite diagnosis were excluded, with 1,213 subjects ultimately being included in the analysis. These 1,213 older adults were aged between 60 and 101, with an average age of (74.79 ± 8.58) at baseline, including 486 males, with an average age of (74.88 ± 8.88), and 727 females, with an average age of (74.73 ± 8.38). By 2020, there had been 447 deaths with mortality at 36.9% (447/1,213), including 198 (40.7%) males and 249 (34.3%) females - the mortality rate of males higher than that of females (*χ^2^ =* 5.273*, P* = 0.022). Of the 1,213 older individuals, 271 had diabetes, with a prevalence of 22.3% (271/1,213) for all genders, 25.1% (122/486) for males and 20.5% (149/727) for females, and without statistically significant difference between the two genders (*χ^2^ =* 3.564, *P* = 0.059); 156 had frailty, with a prevalence of 12.9%, including 43 cases in males (8.8%) and 113 cases in females (15.5%), indicating a higher proportion in females (*χ^2^* = 11.652, *P =* 0.001*);* the prevalence of frailty in older adults with diabetes was 16.6% (45/271), higher than that among those without (11.8%, 111/942) (*χ^2^ =* 4.366, *P =* 0.037). A comparison of baselines for older individuals with or without diabetes and/or frailty showed a higher proportion of the older adults for frailty and diabetes combined with frailty in older adults, females, lower education levels, and widowed older individuals; compared to those not suffering from diabetes or frailty, those suffering from frailty and those with both diabetes and frailty trended towards having 3 or more chronic diseases and taking multiple medications, a significant decrease in activities of daily living (decreased ADL score, increased IADL score) and cognitive function (decreased MMSE score), and increased CES-D score, as well as increased mortality rate (*P <* 0.05 for all). See **Table 1**.

**Table 1:** Comparision of characteristics of the sample as separated by different status of diabetes and frailty.

Variables	No diabetes and no frailty(n=831)	Diabetes and no frailty(n=226)	No diabetes but frailty(n=111)	Diabetes and frailty(n=45)	*F*/*x^2^/H*	*P*-value
Age(x ± s)	73.53 ± 8.08	74.40 ± 8.74	82.27 ± 7.31	82.90 ± 6.12	51.624	<0.001
Sex[n(%)]					14.574	0.002
Men	336 (40.4)	108 (46.6)	28 (25.2)	14 (35.9)		
Women	495 (59.6)	124 (53.4)	83 (74.8)	25 (64.1)		
Education[n(%)]					89.572	<0.001
Primary school	257 (30.9)	72 (31.0)	79 (71.2)	26 (66.7)		
Junior high school	237 (28.5)	74 (31.9)	17 (15.3)	5 (12.8)		
Senior high school or above	337 (40.6)	86 (37.1)	15 (13.5)	8 (20.5)		
Marital Status [n(%)]					44.788	<0.001
Married or cohabiting with spouse	605 (72.8)	159 (68.5)	49 (44.1)	19 (48.7)		
Others^a^	226 (27.2)	73 (31.5)	62 (55.9)	20 (51.3)		
Employment status [n(%)]					3.011	0.390
Working	13 (1.6)	5 (2.2)	0 (0.0)	0 (0.0)		
Retied	818 (98.4)	227 (97.8)	111 (100.0)	39 (100.0)		
≥ 3 types of Chronic Diseases	297 (35.7)	101 (43.5)	64 (57.7)	30 (76.9)	43.708	<0.001
Types of Medication [n(%)]					70.497	<0.001
0	229 (27.6)	60 (25.9)	7 (6.3)	1 (2.6)		
1-3	538 (64.7)	154 (66.4)	75 (67.6)	28 (71.8)		
≥4	64 (7.7)	18 (7.8)	29 (26.1)	10 (25.6)		
ADL Score *[M(Q1,Q3)]*	100 (100,100)	100 (100,100)	90 (65,100)	75 (50,95)	626.120	<0.001
IADL Score *[M(Q1,Q3)]*	0 (0,0)	0 (0,0)	3 (0,8)	5 (3,11)	497.485	<0.001
MMSE Score *[M(Q1,Q3)]*	26 (22,28)	24 (21,26)	23.00 (19.50,24.25)	20 (18,24)	63.496	<0.001
CES-D Score *[M(Q1,Q3)]*	5 (2,8)	5 (2,9)	8.5 (5.0,13.5)	13 (8,21)	147.992	<0.001
FI *[M(Q1,Q3)]*	0.08 (0.05,0.11)	0.09 (0.06,0.13)	0.27 (0.23,0.33	)0.31 (0.24,0.37)	405.930	<0.001
N of Death[n(%)]	259 (31.2)	87 (37.5)	72 (64.9)	29 (74.4)	72.588	<0.001

ADL, Activities of daily living; IADL, Instrumental activity of daily living; MMSE, Mini-mental status examination; CES-D, Center for epidemiologic studies depression scale; FI, frailty index; ^a^Including single, separated, divorce and widowed.

### Influence of diabetes on age-related changes in frailty in the older adults

**Figure 1:**
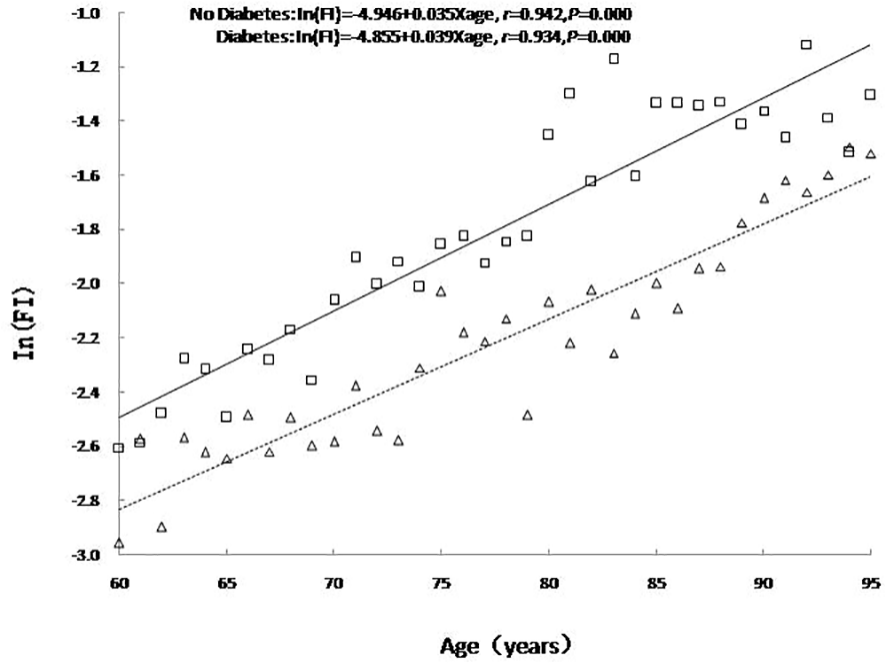
The relationship between age and the mean value of FI. No diabetes: triangle and dashed line; Diabetes: square and solid line.

An analysis of age-related changes to FI values in the older adults with or without diabetes showed that the FI value increased exponentially with age regardless of diabetes status as expressed in the formula In (FI) = A + B× age, including In (FI) = -4.946 + 0.035 × age (*r =* 0.942*, P <*0.001) for older adults without diabetes and In (FI) = -4.855 + 0.039 × age (*r =* 0.934*, P <*0.001) for older adults with diabetes. The FI value was higher for older adults with diabetes than for those without diabetes, i.e., the prevalence of diabetes aggravated the degrees of frailty among the older adults. As the logarithmic coordinates demonstrate, the average annual relative age-related growth rate of health deficits and FI value in older adults with diabetes was higher than in those without diabetes (*β=* 0.039 *vs. β=* 0.035*, t =* 8.367*, P <* 0.001*), i.e.*, the speed of cumulative health deficits was faster for older adults with diabetes than for those without diabetes. See **Figure 1**.

### Influence of diabetes on mortality among older adults with varying degrees of frailty

**Figure 2:**
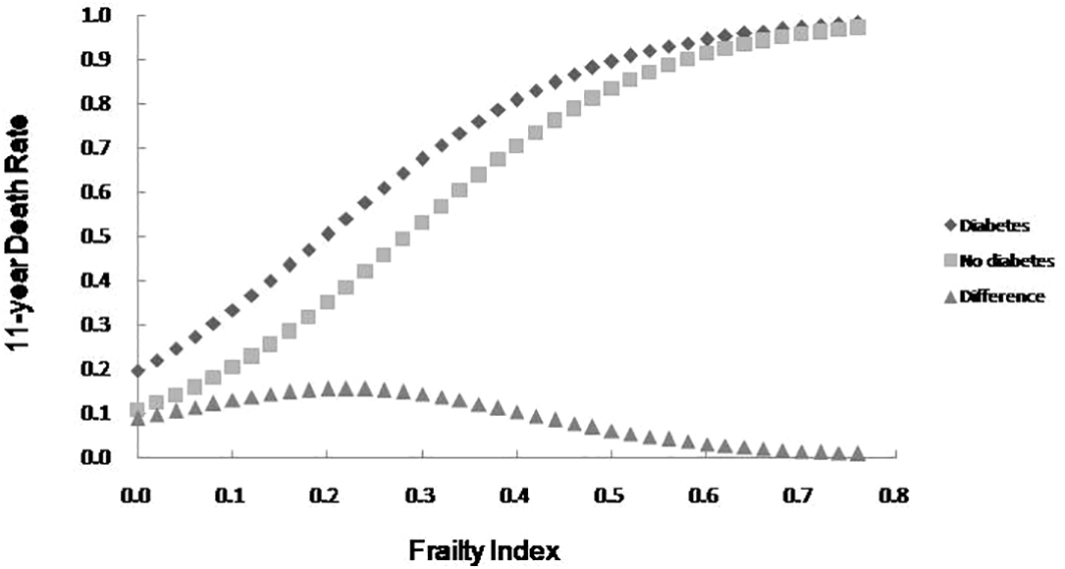
The 11-year death rate as a function of the FI and the mortality difference between older adults with diabetes and no diabetes.

The relationship between FI value and mortality among older adults with or without diabetes was analyzed using a Logistic regression curve according to the literature (17). The results showed that the mortality among older adults with or without diabetes rose with the increase of FI value, and mortality was higher among older adult with diabetes than those without diabetes at any FI value level. An analysis of the difference in mortality rates between older adults with or without diabetes revealed a peak in the range of FI values between 0.1 and 0.3, with the difference diminishing in accordance with increasing frailty. See **Figure 2**.

### Multivariate cox regression analysis of the influence of the presence or absence of diabetes and frailty on mortality risk among the older adults

With death and time to death as dependent variables, and adjusting for variables, including age, gender (Male = 0, Female = 1), education level (Primary high school = 1, Junior high school = 2, Senior high school or above = 3), marital status (Married or cohabiting = 1, Others = 2), it was possible to ascertain from the statistical results that the mortality risk in the older adults increased with age, while the mortality risk in women and older adults who were married or cohabiting was lower than that in the control group. Moreover, compared with the older adults without diabetes and frailty, the highest mortality risk was discovered among those with diabetes and frailty (*HR =* 1.760, 95%*CI:* 1.622 to 1.909, *P <*0.001), followed by those with only frailty (*HR =* 1.594, 95%*CI*: 1.143 to 2.222, *P =* 0.006), and then those with only diabetes (*HR =* 1.475, 95%*CI:*1.238 to 2.766, *P =* 0.033). Furthermore, statistical results by age group revealed that the influence of frailty or diabetes alone on the mortality risk decreased gradually with age, with no statistically significant influence on death in the older adults aged 70- and ≥80 years (all *P >* 0.05). However, the mortality risk among older adults with diabetes and frailty did increase in all age groups (*P <*0.001). See **Table 2**.

**Table 2:** Multivariate Cox regression analysis of the impact of diabetes and frailty on mortality in the older adults of different agegroups.

Variables	Overall(n=1213)			60-(n=385)			70-(n=421)			≥80(n=407)		
	β-value	HR(95%CI)	P-value	β-value	HR(95%CI)	P-value	β-value	HR(95%CI)	P-value	β-value	HR(95%CI)	P-value
Age	0.049	1.050(1.044∼1.057)	<0.001	0.078	1.081(1.053∼1.110)	<0.001	0.045	1.046(1.024∼1.068)	<0.001	-0.003	0.997(0.956∼1.040)	0.896
Sex	-0.324	0.723(0.663∼0.788)	<0.001	-0.483	0.617(0.520∼0.731)	<0.001	-0.309	0.734(0.646∼0.834)	<0.001	-0.145	0.865(0.734∼1.020)	0.085
Education	-0.050	0.951(0.885∼1.022)	0.170	-0.106	0.900(0.788∼1.027)	0.118	-0.080	0.923(0.831∼1.025)	0.136	-0.099	0.906(0.855∼1.276)	0.179
Marital Status	0.310	1.363(1.211∼1.533)	<0.001	0.313	1.368(1.149∼1.628)	<0.001	0.234	1.263(1.054∼1.515)	0.012	0.455	1.576(1.075∼2.310)	0.020
Status of diabetes and frailty^a^												
Diabetes and no frailty	0.389	1.475(1.238∼2.766)	0.033	0.275	1.617(1.120∼4.654)	0.018	0.478	1.512(0.667∼3.897)	0.289	0.586	1.497(0.444∼7.268)	0.411
No diabetes but frailty	0.466	1.594(1.143∼2.222)	0.006	0.626	1.871(1.150∼3.044)	0.012	0.499	1.647(0.949∼2.857)	0.076	0.142	1.152(0.511∼2.599)	0.733
Diabetes and frailty	0.565	1.760(1.622∼1.909)	<0.001	0.657	1.929(1.644∼2.262)	<0.001	0.602	1.826(1.624∼2.054)	<0.001	0.403	1.496(1.284∼1.743)	<0.001

FI,frailty index; ^a^ No diabetes and no frailty as a reference.

### Comparison of survival curves for older adults with or without diabetes and frailty

**Figure 3:**
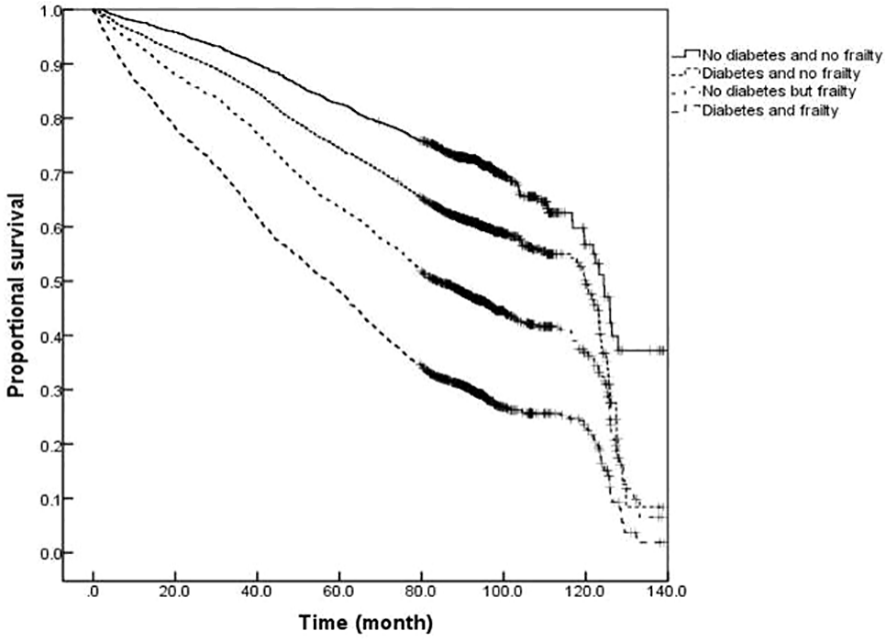
Kaplan-Meier curves for the proportional survival of total population with different diabetes and frailty status.

**Figure 4:**
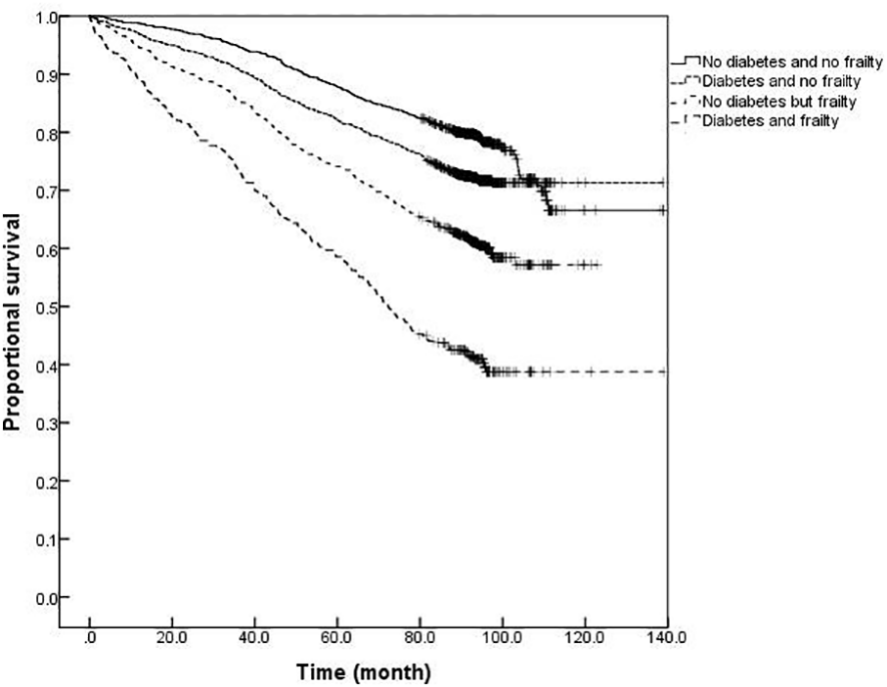
Kaplan-Meier curves for the proportional survival of older adults aged 60- years with different diabetes and frailty status.

**Figure 5:**
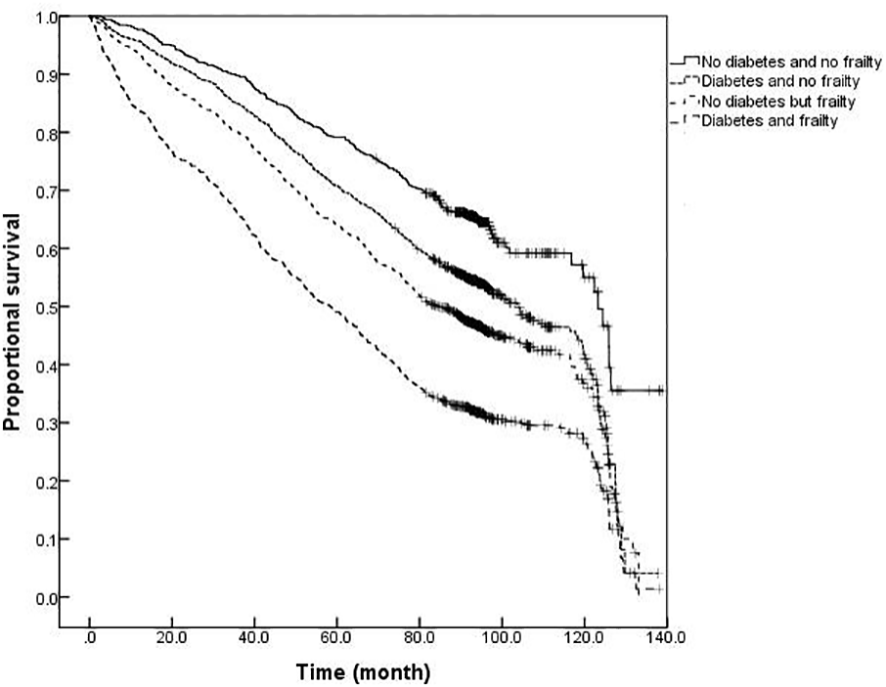
Kaplan-Meier curves for the proportional survival of older adults aged 70- years with different diabetes and frailty status.

**Figure 6:**
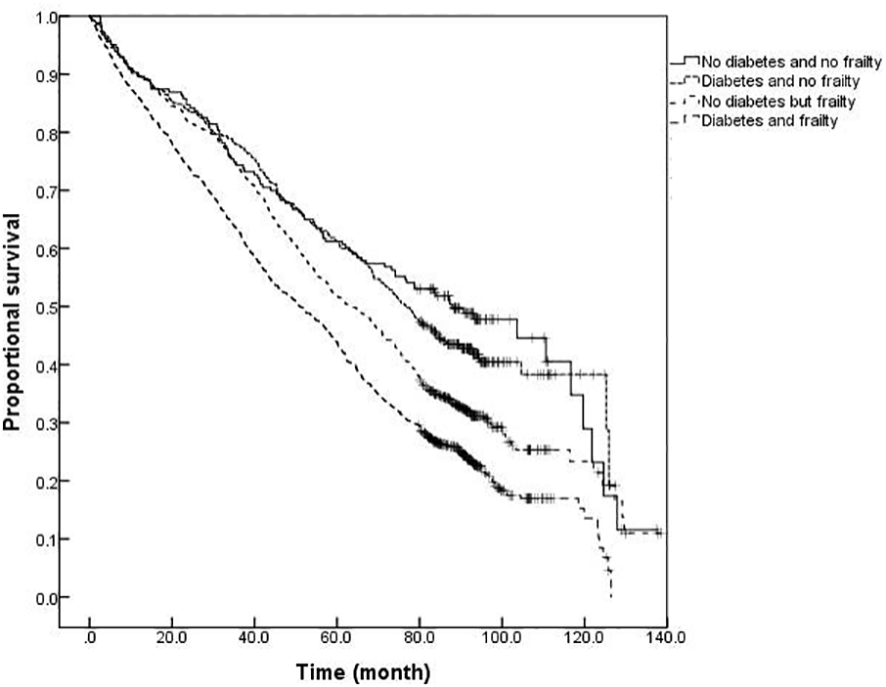
Kaplan-Meier curves for the proportional survival of older adults aged ≥80 years with different diabetes and frailty status.

The survival analysis showed that the median survival of those suffering from diabetes and frailty to be the shortest at just 57.23 (95% *CI:* 54.05 to 60.41) months, lower than the 83.78 (95% *CI:* 79.33 to 88.23) months in those suffering from frailty alone, and 119.93 (95% *CI:* 113.84 to 126.02) months in those with only diabetes, and 124.39 (95% *CI:* 119.76 to 129.02) months in the older adults with neither diabetes nor frailty (*P <*0.001). A further comparison of survival curves of older adults s with or without diabetes and frailty in different age groups revealed that the survival rates of older adults in the 60-, 70-, and ≥80-year-old age groups decreased with the prevalence of diabetes and frailty (all *P<*0.001). The results of a pairwise comparison of the survival rate of older adults with different diabetes and frailty status in all age groups showed statistically significant differences, only except for the survival rate between those with only diabetes and with neither diabetes nor frailty in the ≥ 80-year-old age group *(P =* 0.346). See **Figures 3**–**6**.

## Discussion

Frailty and diabetes are two important health problems associated with aging in older adults. Meanwhile, both conditions frequently co-occur and are increasingly prevalent among older adults. The results of this study revealed that the prevalence of diabetes among older adults in Beijing was 22.3%, which was consistent with the results of the Chinese diabetes survey. In the survey by Li et al. (2) and Wang et al. (18), the prevalence of diabetes in the Chinese population aged 60 years or more was 20.9% and 30.0% in 2013 and 2017, respectively, with an awareness rate of approximately 30.0%. Comparison at baseline in this study showed that older adults, females, individuals with lower education levels, and widowed older adults were associated with a higher proportion of frailty and diabetes with frailty. Among older adults with diabetes and frailty, there was an increased proportion with 3 or more chronic diseases and multiple medications, a significant decrease in the activities of daily living and cognitive functions, and an increase in depression scores, all of which were consistent with previous studies (19–21). Therefore, targeted intervention would be desirable in the management of older adults with diabetes and frailty to delay the course of diseases, relieve harm resulting from comorbidities, reduce the risk of adverse outcomes, and improve the level of diabetes management overall.

The results of this study revealed that the prevalence of frailty in older adults with diabetes was 16.6%, which was higher than in those without diabetes (11.8%). Moreover, the FI value of older adults with diabetes was higher than that of those without diabetes at any age, *i.e.*, the prevalence of diabetes aggravated the degrees of frailty in older adults. As the logarithmic coordinates demonstrate, the average annual relative growth rate of health defects and FI value over age in older adults with diabetes was higher than among older adults without diabetes (*β*= 0.039 *vs. β*= 0.035), *i.e.*, the speed of cumulative health defects was greater when diabetes was present. As reported in the study of Kong et al. (6), the overall prevalence of older adults with diabetes experiencing conditions of frailty and pre-frailty in Chinese communities was 20.1% and 49.1%, respectively, with older adults suffering from diabetes more likely to develop frailty than those without diabetes (*OR* = 1.61, 95% *CI*: 1.47-1.70, *P* < 0.001). The Beijing Longitudinal Study of Aging II (BLSA-II) (22) revealed that the prevalence and incidence of frailty in older adults with diabetes were significantly higher than in those without diabetes (19.32% *vs.*11.92%, and 12.32% *vs.* 7.04%). Patients with diabetes at 65 years or older are more prone to frailty than those without diabetes. Angulo et al. (23) claimed the co-occurrence of diabetes and frailty in older adults not to be surprising as both age-related conditions share a common underlying pathophysiological mechanism, which may include premature aging of the organ system in a hyperglycemic state, chronic inflammation, increased oxidative stress, accumulation of advanced glycation end products, and insulin resistance (24). In recent years, some achievements have been made in the exploration of the common mechanism of diabetes and frailty at various levels of genes, protein molecules, cells, tissues, and organs, mainly including insulin resistance, arteriosclerosis, chronic inflammation, oxidative stress, cell damage, and mitochondrial dysfunction among other theories. For example, C-reactive protein and interleukin-6, typical inflammatory factors, were present at a high level in patients with diabetes and frailty (25). Amino acid metabolism disorders may be a common pathway for dysfunction in patients with diabetes and frailty. Calvani et al. (26) investigated the amino acid metabolism profile of older adults with diabetes and frailty and discovered that the levels of some characteristic metabolites such as serum 3-methylhistidine were higher. A structural magnetic resonance study found that, in patients with diabetes and frailty, the decreased size of gray matter involved in motor control was linked to decreased muscle size and strength (27). In addition, metabolites of the intestinal microbiota and peripheral inflammation affected the decomposition and synthesis of muscle proteins through various signal pathways regulated by inflammation and insulin sensitivity, which also indirectly impacted food intake, resulting in decreased protein synthesis and body frailty (28).

The results of this study also demonstrated that mortality was higher among older adults with diabetes than those without diabetes at any degree of frailty. The difference in mortality between older adults with or without diabetes peaked in the range of FI values between 0.1 and 0.3, with the difference gradually narrowing with an increasing degree of frailty. In order to further illustrate the influence of diabetes and frailty on the mortality risk, the Cox regression analysis was performed in this study after adjusting for confounding factors, such as age, gender, and education level, and the results revealed the highest mortality risk among older adults with diabetes and frailty. Further statistical results by age group presented that the mortality risk for older adults with diabetes and frailty was increased in all age groups and exerted a greater influence on the mortality risk than for those suffering from only frailty or diabetes. A follow-up study found frailty to be helpful when seeking to identify diabetic patients at high risk of mortality (29). Patients with diabetes and frailty also suffered high rates of hospitalization and all-cause mortality (30). Combined with the results of this study, it is suggested that healthcare professionals should pay greater attention to the screening and assessment of frailty conditions in older adults with diabetes, especially among those with pre-frailty or mild frailty (FI value: 0.1- 0.3) who can receive more benefit. Japanese scholars have suggested that the management of older adults with diabetes should shift its focus from the prevention of metabolic syndrome to the prevention of frailty (31). It has also been proposed that the assessment of frailty should be conducted in all older adults with diabetes as early identification of frailty, assessment of its degree, and timely intervention can greatly delay the progression of diabetes and related complications in the management of diabetes (32). At present, the frailty assessment scale for the general population is still used in the assessment of frailty in individuals with diabetes, while the FI model was adopted to assess the frailty of the older adults in this study. The FI model is currently one of the most commonly used methods for the assessment of frailty in the older adults, and several studies have confirmed its satisfactory reliability and validity (33, 34). Additionally, the results of the systematic review study have suggested that FI is the only assessment tool capable of covering all frailty-related factors, and FI is also the most useful assessment tool for frailty in conventional care and community settings (34). Consequently, we employed the FI model to assess the frailty of the older adults in the community. Furthermore, the Comprehensive Frailty Assessment Instrument (CFAI) and the Tilburg Frailty Index have both been recommended for the screening of early frailty in the older adults with chronic diseases such as diabetes and hypertension in primary care institutions, and at the same time, the Frailty Index (FI-CGA) based on the Comprehensive Geriatric Assessment can better assess the comprehensive conditions of hospitalized patients and quantify the degrees of frailty, all of which are of great significance in guiding diabetic patients in blood glucose control and drug selection in relation to degrees of frailty. As regards the management of older adults with diabetes and frailty, studies have disclosed that frailty, as an unfavorable factor causing severe hypoglycemia in diabetic patients experiencing intensive glycemic control treatment, is capable of compromising the efficacy of intensive treatment. For instance, Nguyen et al. (8) observed patients with type 2 diabetes who received intensive glucose-lowering therapy and found that the incidence of severe hypoglycemia in patients with frailty was significantly higher than that in those without frailty. Hence, the Expert Consensus Statement on the Management of Older Adults with Type 2 Diabetes recommends that the glycosylated hemoglobin (HbA1c) be controlled at 6%-7.5% in healthy and mild frailty patients, appropriately relaxed to 8.0% in moderate frailty patients, and not more than 9.0% in severe frailty patients, such as loss of independence or a combination with serious complications (32). Besides, the American Diabetes Association Professional Practice Committee (35) recommends that the target value of HbA1c control be relaxed to 8.0% in patients with varying degrees of functional dependence, and overall, a looser target value of glucose control is suggested for patients with diabetes and frailty after sufficient assessment of individual conditions (35). Furthermore, the results of the survival analysis in this study indicated that patients with diabetes and frailty suffered the shortest survival time, followed by those only with frailty, those only with diabetes, and those without diabetes and frailty, which further verified the conclusion of the regression model for the mortality risk.

The following limitations of this study should be noted. Baseline data was acquired based on a questionnaire survey and some key confounders (such as comorbidities) were not included, with a potential for information bias. In addition, as a prospective study, older adults lost to follow-up were relatively young individuals who had left or moved away from the place of the survey, and the possible loss to follow-up bias may have a certain impact on the study results. Moreover, the causes of death in the older adults were not collected in this study, and the impact of other causes of death on the study results cannot be excluded, so it would be necessary to further improve the questionnaire and supplement related information for a more profound analysis in the future.

## Conclusions

Frailty is common among older adults suffering from diabetes, and there is an increased risk of poor health outcomes, such as death, among older adults suffering from diabetes and frailty. Given the interaction between diabetes and frailty, it would be advisable to strengthen our knowledge of frailty, promote the assessment of frailty, identify it early, and apply targeted interventions during the diagnosis and management of older adults with diabetes, so as to avoid or postpone the adverse effects caused by frailty and ease the medical burden.

## Data availability statement

The original contributions presented in the study are included in the article/**Supplementary Material**. Further inquiries can be directed to the corresponding authors.

## Ethics statement

Written informed consent was obtained from the individual(s) for the publication of any potentially identifiable images or data included in this article.

## Author contributions

JS conducted the survey, performed statistical analysis the data and drafted the paper. YT and LW helped with the analysis and assisted with manuscript preparation. SC, ZZ, LM and BZ conducted the survey and collected the data. CD assisted data analysis and result interpretation. HX and PY initiated and designed the study, revised the paper and finally approved the version to be published. All authors contributed to the article and approved the submitted version.
