# Supplementary material for: Combined effect of diabetes and frailty on mortality among Chinese older adults: A follow-up study
Source: Front Endocrinol (Lausanne). 2023 Jan 16;13:1105957. doi: 10.3389/fendo.2022.1105957 (PMC9884703; doi:10.3389/fendo.2022.1105957)
Supplement: Supplementary file 1 [file Table_1.docx]

**Appendix 1** **Variables for Frailty Index Construction and Their Codes**

| **Variables** | **Codes** |
| --- | --- |
| **Comprehensive geriatric assessment** |  |
| Falls | No=0, Yes=1 |
| Urinary incontinence | Never=0, 1 time/week or less=0.25, 2-3 times/week=0.5, About 1 time/day=0.75, Several times/ day=1 |
| Pain | None=0, Tolerable without affecting any activities=0.33, Tolerable but affecting some activities=0.66, Intolerable but still able to make phone calls/watch TV/perform other activities=1 |
| Constipation | No=0, Yes=1 |
| Weight loss | No=0, Yes=1 |
| Sleep disorder | No=0, Yes=1 |
| Usage of sleep aids | No=0, Yes=1 |
| **Visual and hearing assessment** |  |
| Vision | > 4 m=0, 1-3 m=0.5, 1 m or less=1 |
| Hearing | Completely clear=0, Not very clear= 0.5, Not clear at all = 1 |
| **Walking balance function** |  |
| Usage of walking aids | No=0, Yes=1 |
| Walking 400 m independently | Yes=0, No=1 |
| Static balance test | > 10s=0, ≤10s=0.5, Unable to complete=1 |
| Dynamic balance test | > 10s=0, ≤10s=0.5, Unable to complete=1 |
| 5 times sit-to-stand test | <10s=0, ≥10s=0.5, Unable to complete=1 |
| Up-and-go test | <12s=0, ≥12s=0.5, Unable to complete=1 |
| **Diseases and medication** |  |
| Chronic diseases(14 types)^a^ | Each no = 0, Each yes = 1 |
| Number of medications | Infrequent medications = 0, 1-3 medications = 0.5, ≥4 = 1 |
| **Assessment of activities of daily living** |  |
| ADL | 100 points=0, 75-95 points=0.25, 50-70 points=0.5, 25-45 points=0.75, 0-20 points=1 |
| IADL | ≤5 points=0, > 5 points=1 |
| **Cognition and emotion** |  |
| Memory loss | No=0, Yes=1 |
| Emotional instability | Never=0, Sometimes=0.5, Often=1 |
| MMSE^b^ | Cut-off value is normal = 0, Below cut-off is a cognitive deficit=1 |
| **Depression assessment** |  |
| CES-D | < 10 points=0, ≥ 10 points=1 |

Note: ADL: activities of daily living, IADL: instrumental activities of daily living, MMSE: mini-mental state examination; CES-D: Center for Epidemiological Studies-Depression Scale (simplified version); ^a^The 14 types of chronic diseases include hypertension, heart disease, anemia, hyperlipidaemia, sleep apnea syndrome, gastrointestinal disease, cerebrovascular disease, dementia, tumour, protrusion of intervertebral disc, thyroid disease; osteoporosis, osteoarthritis, arthrolithiasis (except for diabetes); ^b^MMSE cut-off value is related to the level of education, 17 points for no education, 20 points for less than 6 years of education, 24 points for more than 6 years
